# Validation of real-time polymerase chain reaction versus conventional polymerase chain reaction for diagnosis of brucellosis in cattle sera

**DOI:** 10.14202/vetworld.2021.144-154

**Published:** 2021-01-19

**Authors:** Nour H. Abdel-Hamid, Eman I. M. Beleta, Mohamed A. Kelany, Rania I. Ismail, Nadia A. Shalaby, Manal H. M. Khafagi

**Affiliations:** 1 Department of Brucellosis Research, Animal Health Research Institute, Agricultural Research Center, Dokki, Giza 12618, Egypt; 2 Department of Microbiology, The Central Laboratory of Residue Analysis of Pesticides and Heavy Metals in Food, Agricultural Research Center, Dokki, Giza, Egypt; 3 Department of Parasitology and Animal Diseases, National Research Centre, 33 Bohouth St., Dokki, Giza, 12622, Egypt

**Keywords:** bacteriological results, *Brucella*, conventional polymerase chain reaction, diagnostic sensitivity, diagnostic specificity, TaqMan real-time-polymerase chain reaction

## Abstract

**Background and Aim:**

Different polymerase chain reaction (PCR) techniques have and are still being used for the direct detection of *Brucella* DNA in serum samples of different animal species and humans without being validated or properly validated, resulting in discrepancies. Thus, this study aimed to evaluate the diagnostic performance of the TaqMan Real-Time-PCR (RT-PCR) targeting the bcsp31 gene versus conventional PCR for the accurate diagnosis of brucellosis at the genus level in cattle sera.

**Materials and Methods:**

One hundred and eighty-four serum samples were collected from bacteriologically positive and negative cows with ages ranging from 1 to 5 years old at some infected private farms in the Nile Delta under quarantine measures as well as brucellosis free farms. These samples were classified into four groups after serological diagnosis and investigated by TaqMan RT-PCR and conventional PCR targeting the IS711 gene for *Brucella* DNA detection. The diagnostic performance characteristics of both PCR techniques were estimated considering the bacteriological results as a gold standard.

**Results:**

TaqMan RT-PCR revealed superiority over conventional PCR; it was able to detect *Brucella* DNA in 95% (67/70) and 89% (25/28) of the cattle sera samples belonging to Group 1 (serologically and bacteriologically positive) and Group 2 (serologically negative but bacteriologically positive), respectively. On evaluating the diagnostic performance, TaqMan RT-PCR showed superior diagnostic sensitivity (93.9%), diagnostic specificity (88.4%), performance index (182.3), almost perfect kappa agreement (0.825±0.042), strong positive correlation (r=0.826), high accuracy based on the receiver operating characteristic (ROC) curve, and area under the ROC curve (0.911) at p<0.05 and CI of 95%.

**Conclusion:**

A cattle serum sample is not the metric of choice for targeting *Brucella* genomic DNA by conventional PCR. The time-saving and rapid TaqMan RT-PCR method revealed a better diagnostic performance in the detection of *Brucella* DNA in cattle sera. Such performance offered by TaqMan RT-PCR may be considered a step toward the possibility of using such technology in the direct differentiation between *Brucella*-infected and -vaccinated cattle immunized by smooth vaccines from cattle sera using primers specific for such vaccines.

## Introduction

Brucellosis is a zoonotic disease that affects a wide range of domestic, wild, and marine mammals and causes reproductive disorders, including abortion, retained placenta, stillbirth, and orchitis as well as decreased milk yield and, less frequently, arthritis. At present, 11 species have been identified [1]. Among them, three species have been reported in Egypt; *Brucella melitensis* isolated from ruminants, Nile catfish, and humans; *Brucella abortus* isolated from cattle, dogs, and cats; and *Brucella suis* has been recovered from cattle and swine [2-4]. The identification of the Brucellae is done through the direct detection of *Brucella* organisms in milk, aborted material, lymph nodes, and other tissues through the isolation and typing of the causative microorganism or indirectly using serological tests. None of the serological tests used in the diagnosis of brucellosis can be used exclusively in all of these animal species. These tests are not 100% reliable and have limitations under all epidemiological circumstances. Subsequently, the seropositive samples identified by screening tests (such as the Rose Bengal plate test [RBPT]) shall be confirmed by the complement fixation test (CFT) that is recommended by the OIE for the contribution of disease eradication [1]. Several molecular methods, including real-time polymerase chain reaction (RT-PCR), have been developed that take into account the high DNA homology characteristic to the genus *Brucella*. This development has coincided with the identification of different *Brucella* genome regions, facilitating, to a certain extent, the differentiation of *Brucella* species and some of their biovars, where it provides an extra means of direct detection and complementary identification and typing methods of *Brucella* spp. [5].

The diagnostic performance of conventional PCR and RT-PCR is affected by different factors such as the DNA extraction method, type of fluorogenic-labeled probe in case of RT-PCR, and the presence of foreign DNA and inhibitors in the samples [6]. Despite its high speed and diagnostic sensitivity (DSe) and specificity [7], the presence of inhibitors may decrease the sensitivity of PCR methods [8]. RT-PCR methods have been improved using TaqMan fluorogenic-labeled probes that exploit the 5′ nuclease activity of Taq DNA polymerase. These fluorogenic probes allow for the improvement of real-time diagnostic performance by detecting only specific products and avoiding the detection of accumulated nonspecific PCR products, which is the biggest issue contributing to the decreased specificity of SYBR green-based RT-PCR [9]. The PCR techniques (conventional PCR and RT-PCR) used for the detection of *Brucella* genomic DNA in serum samples require validation regardless of the DNA released into the bloodstream during bacteremia [10] and significantly lower levels of PCR inhibitors in serum samples [11], particularly after variations and discrepancies in the diagnostic performance of such techniques have been reported.

Thus, this study aimed to evaluate the diagnostic performance of TaqMan based RT-PCR and conventional PCR for the accurate diagnosis of brucellosis in cattle sera.

## Materials and Methods

### Ethical approval

The research ethics committee for experimental and clinical studies, Animal Health Research Institute (No. 165623), has approved the protocol of this study. That is aligned with the guidelines laid down by the Egyptian Network of Research Ethics Committees and is compatible with the international laws and regulations concerning the ethical considerations in research. Efforts were taken into account to minimize animal pain or discomfort and to decrease the number of animals required for this study.

### Study period and location

Ninety-eight selected serum samples were collected from cattle reared in *Brucella* infected private farms under quarantine measures in Kafrelsheikh, Sharqia, and Dakahlia governorates, Nile Delta. Added to the above, 86 serum samples were collected from private farms in Damietta (n=30) with no history of brucellosis infection for the last three years and imported cattle serum samples from Germany (n=56). Serum samples were collected during the period from February 2018 to March 2019.

### Experimental design

#### Sampling and design of groups

Kindly, 98 serum samples were selected out of 4500 serum samples from the serum bank of Brucellosis Research Department, Animal Health Research Institute, to validate RT-PCR and conventional PCR. These samples were collected from infected cows of ages ranging from 1 to 5 years old at private farms under quarantine measures located in certain Nile Delta governorates. *B. melitensis* biovar 3 has been recovered from animals that belong to these infected farms. Besides, 86 bacteriologically negative samples were selected from the same bank in addition to the above 98 serum samples for the same validation aim. These selected serum samples (n=184) of bacteriologically positive and negative animals were serologically diagnosed using the RBPT for screening, followed by the CFT as confirmation (British version). Serum samples were interpreted as positive if they revealed a positive reaction to RBPT and/or CFT.

Four groups were created based on the results of the serological and bacteriological examinations. Group 1 (n=70) includes serologically and bacteriologically positive samples. Group 2 (n=28) includes bacteriologically positive but serologically negative serum samples. Group 3 (n=30) contains serologically positive but bacteriologically negative serum samples. Finally, Group 4 (n=56) includes samples that tested negative on both serological and bacteriological tests.

The serum samples of Group 4 (n=56), were obtained from brucellosis-free farms with no history of brucellosis infection.

### Tests and procedures

#### Serological tests

Serum samples were serologically examined against brucellosis using Rose Bengal (RBT 8%), and complement fixation (CFT). Rose Bengal antigen was purchased from National Veterinary Services Laboratories/Diagnostic Bacteriology Laboratory, USDA, USA. The RBPT was performed according to protocols cited elsewhere [12]. Any visible agglutination recorded within 4 min was considered positive for the RBPT. The British CFT antigen was purchased from APHA, New Haw, Addlestone, Surrey KT15 3NB, UK. The British version of CFT was done as per the previous study [13]. The results of CFT were considered positive at a titer of 1/4 (50% fixation), equivalent to ≥20 ICFTU/mL.

#### DNA extraction

DNA extraction from the serum samples was done with the aid of a Quick-DNA^™^ Universal Kit (The Epigenetics Company, USA), with an amendment of the manufacturer’s recommendations. A 200 μL volume of each serum sample was incubated with 20 μL of proteinase K, 200 μL of biofluid, and cell buffer at 55°C for 10 min. After incubation, 420 μL of genomic binding buffer was added to the lysate. The mixture was transferred through a Zymo-Spin^™^ IIC-XL column (Epigenetics, USA) into a collection tube. The sample was then washed and centrifuged according to the manufacturer’s recommendations. Nucleic acid was eluted with 50 μL of elution buffer provided with the extraction kit.

### Conventional PCR

#### Oligonucleotide primers

The primers were supplied from Biobasic (Canada) and are listed in Table-1.

**Table-1:** Primers sequences, target gene, amplicon sizes, and cycling conditions for conventional PCR.

Target gene	Target agent	Primers sequences	Amplified segment (bp)	Primary denaturation	Amplification (35 cycles)			Final extension	Reference

					Secondary denaturation	Annealing	Extension		
1S711	*Brucella* genus	IR1 GGC-GTG-TCT-GCA-TTC-AAC-G IR2 GGC-TTG-TCT-GCA-TTC-AAG-G	839	94°C 5 min	94°C 30 s	55°C 40 s	72°C 50 s	72°C 10 min	[14]

PCR=Polymerase chain reaction

#### PCR amplification [14]

The primers were utilized in a 25-μL reaction containing 12.5 μL of Emerald Amp Max PCR Master Mix (Takara, Japan), 1 μL of each primer at a 20 pmol concentration, 4.5 μL of water, and 6 μL of DNA template. The reaction was performed on an Applied Biosystem 2720 thermal cycler.

#### Analysis of the PCR products

The PCR products were separated by electrophoresis on a 1% agarose gel (Applichem, Germany, GmbH) in 1× TBE buffer at room temperature using a current of 5 V/cm. For gel analysis, 15 μL of the products were loaded in each gel slot. A 100-bp DNA ladder (Fermentas, Thermo, Germany) was used to determine the fragment sizes. The gel was photographed using a gel documentation system (Alpha Innotech, Biometra), and the data were analyzed using computer software.

#### TaqMan based RT-PCR

TaqMan RT-PCR was done on serum samples using primers and probes [15] targeting the bcsp31 gene (GenBank accession number M20404). Primers’ sequences, target gene, and TaqMan probe are listed in Table-2.

**Table-2:** Primers sequences, target gene, and cycling conditions for TaqMan RT-PCR.

Target gene	Target agent	Primers sequences	Amplified segment (bp)	Primary denaturation	Amplification (40 cycles)			Dissociation curve (1 cycle)		
	
					Secondary denaturation	Annealing (Optics on)	Extension	Secondary denaturation	Annealing	Final denaturation
BSCP31	*Brucella* genus	BSCP31 Forward primer GCTCGGTTGCCAATATCAATGC	150	94°C 5 min	94°C 15 s	55°C 30 s	72°C 45 s	94°C 1 min	55°C 1 min	94°C 1 min
	BSCP31 Reverse primer GGGTAAAGCGTCGCCAGAAG								
	RT-PCR probe AAATCTTCCACCTTGCCCTTGCCATCA-FAM/BHQ1								

RT-PCR=Real-time polymerase chain reaction

#### PCR amplification

TaqMan RT-PCR

The primers were used in a 25-μL reaction containing 12.5 μL of the HERA q-PCR PCR Master Mix (Willowfort, UK), 0.2 μL of each primer at a 20 pmol concentration, 0.1 μL of the probe, 7.0 μL of DNase free water, and 5 μL of DNA template. The reaction was performed in a Thermo Scientific Piko RT-PCR machine (Thermo Fisher Scientific, US). The DNA of *Brucella*-positive and -negative controls were included in each run to indicate any amplicon contamination or amplification failure.

### RT-PCR standard curves

Standard curves were created by plotting the cycle threshold (CT) values of the RT-qPCR performed on ten-fold serially diluted purified DNA extracted from ten-fold serial dilutions of the *B. melitensis* reference strain Ether (ATCC 23458) in sterilized serum samples (10×10^5^-10×10^1^ CFU/mL) against the log input cells/mL [16] The ten-fold serial dilutions of a known amount of *B. melitensis* reference strain Ether purified DNA was tested in triplicate.

### Analysis of the TaqMan rt-PCR results

Amplification curves and CT values were determined by the Stratagene MX3005P software (Agilent, Santa Clara, USA).

### Statistical analysis

The diagnostic performance parameters of PCR and RT-PCR were estimated, considering bacteriological isolation and typing as the gold standard, using DSe, diagnostic specificity (DSp), Kappa agreement, receiver operating characteristics (ROC), area under the ROC (AUC) curve, positive predictive value (PPV), and negative predictive value (NPV) at p≤0.05 with a 95 % confidence interval (CI). These parameter values were estimated using SPSS Statistics for Windows, version 21.0. (IBM Corp., Armonk, N.Y., USA). While the remaining diagnostic performance parameters (performance index and accuracy %) were calculated, according to the method described elsewhere [17,18].

## Results and Discussion

Validation is the process that determines the fitness of a developed, standardized, and optimized assay used for the aim of diagnosis. Conventional PCR and RT-PCR have been used as a diagnostic tool in most of the published research for the detection of DNA of *Brucella* species in serum samples of small ruminants [19], camels [20,21], bovine [22-24], swine [25], and humans [26,27] without first being validated or validated in an accurate manner. Some authors have previously considered serum as the sample of choice for the same purpose [28]. Most of the assays that have been used in the diagnosis of brucellosis were standardized primarily for use in cattle. Moreover, there has been limited validation of the PCR techniques used for direct diagnosis, as reported by OIE [1]. In addition, PCR techniques are still not recommended by the OIE for the declaration of an individual animal or population to be free from *Brucella* infection. It is also not deemed suitable to be used effectively in eradication policies or the estimation of a herd’s disease prevalence [1].

Validation includes estimates of the analytical and diagnostic performance characteristics of a test [29]. Bacteriological typing of *Brucella*, the gold standard, is the only unequivocal definitive diagnosis of brucellosis [1,30]. In an effort to develop a validated technique, 184 serum samples (2% error, 95% CI) were selected from culture-positive and -negative animals as negative and positive reference samples. These samples were used to estimate the diagnostic performance parameters of RT-PCR and conventional PCR targeting *Brucella* genomic DNA from serum samples of cows. Validation was performed according to the principle and methods of validation of diagnostic assays for infectious diseases [29].

**Figure-1:**
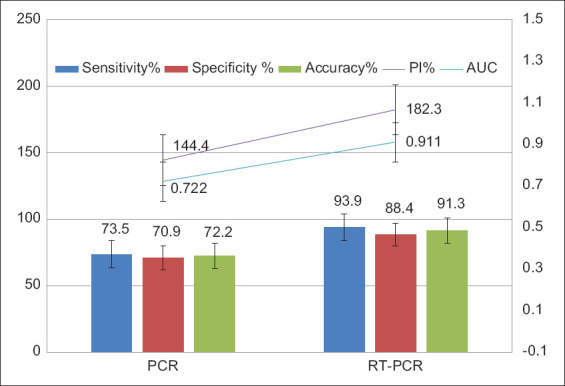
Diagnostic sensitivity, specificity, performance indices, accuracy, and area under the curve of real-time polymerase chain reaction (PCR) versus conventional PCR targeting Brucella DNA in cattle sera.

The DSe (proportion of positive test results among diseased; DSe and DSp (proportion of negative test results among the healthy; Dsp) were calculated for both RT-PCR and conventional PCR; the results are tabulated in Table-3 and Figure-1. RT-PCR offers a better DSe (93.9%) and DSp (88.4%) over conventional PCR (DSe=73.5%; DSp=70.9%). The improved DSe and specificity of RT-PCR match the results reported by Zeybek *et al*. [7] and AL-Ajlan *et al*. [31]. On the contrary, these findings are in disagreement with the results reported by Tiwari *et al*. [32] and Dal *et al*. [33].

**Table-3:** Kappa agreement and the diagnostic performance parameters of RT-PCR versus PCR techniques targeting *Brucella* genomic DNA in cattle sera.

Statistical parameters	RT-PCR				PCR			
	
	TP	FP	TN	FN	TP	FP	TN	FN
		
	92	10	76	6	72	25	61	26
Sensitivity % (SPSS)	93.9%				73.5%			
Specificity % (SPSS)	88.4%				70.9%			
Performance indices (PI)=(Se+Sp)	182.3				144.4			
** Kappa agreement±standard errors (SPSS)	0.825±0.042				0.444±0.066			
AUC (SPSS)	0.911				0.722			
Accuracy= (TP+TN)/(TP+TN+FP+FN)	91.3%				72.2%			
Positive predictive value (PPV) TP/(TP+FP)	0.9				0.74			
Negative predictive value (NPV) TN/(TN+FN)	0.92				0.7			

AUC=Area under the ROC curve representing a single parameter of accuracy at confidence interval of 95%.

** agreement (±Standard error) with bacteriological examination at p<0.05 and 95% confidence interval, PPV=Positive predictive value, NPV=Negative predictive value, TP=True positive, TN=True negative, FP=False positive and FN=False negative. Se=Sensitivity, Sp=Specificity, RT-PCR=Real-time polymerase chain reaction

**Figure-2:**
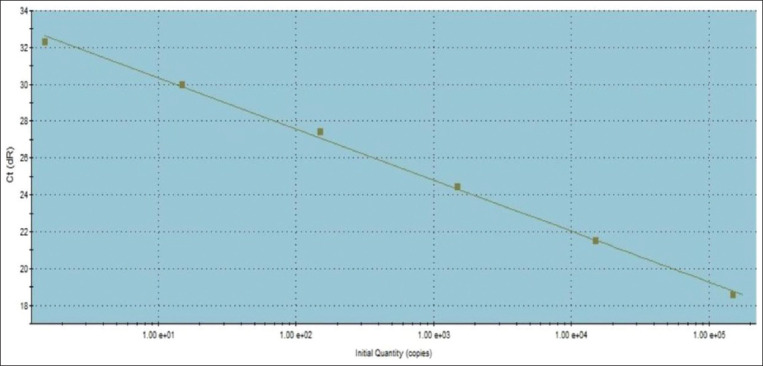
Standard curve of TaqMan real-time polymerase chain reaction targeting *Brucella* bscp31 gene plotting cycle threshold values versus log template concentrations.

**Figure-3:**
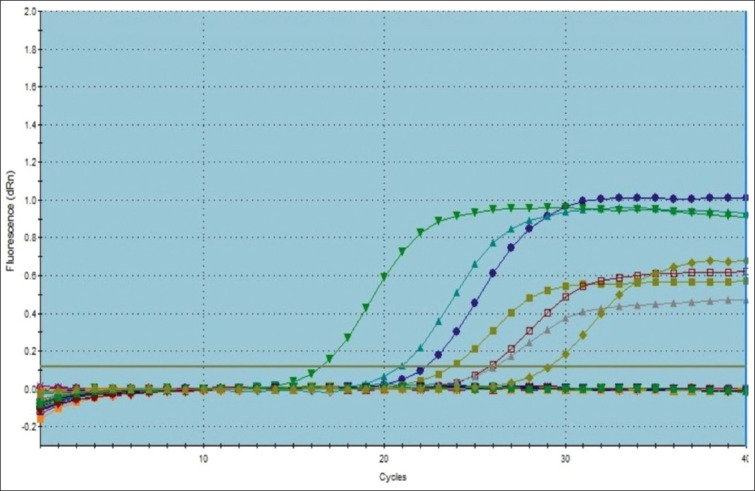
Amplification curves of TaqMan real-time polymerase chain reaction for the detection of the DNA of the *Brucella* genus in cattle sera.

**Figure-4:**
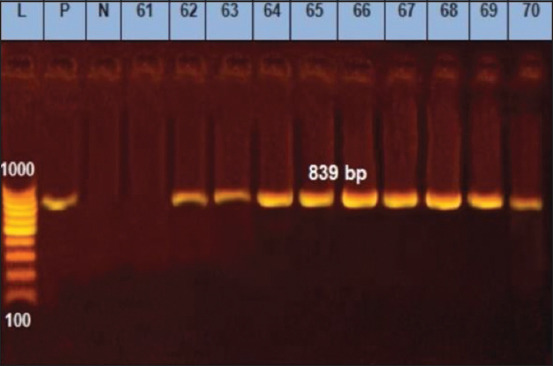
Detection of IS711 gene specific for genus *Brucella* by conventional polymerase chain reaction (PCR). Lane 1, PCR marker; Lane 2(P), *B. melitensis* reference strain Ether; lane3 (N), control negative; Lane 4 (sample 61), negative sample; lane 5-13 (samples No. 62, 63, 64, 65, 66, 67, 68, 69, 70), *Brucella* positive samples.

The better combined DSe and DSp of RT-PCR may be attributed to its unique design based on the oligonucleotide double-labeled probe that relies on the transfer of light energy between two adjacent dye molecules, a process referred to as fluorescence resonance energy transfer, and the exonuclease activity of the Taq polymerase that enables the detection of only specific amplification products [9]. In addition, the TaqMan based RT-PCR has a low DNA limit of detection (≥10^2^ CFU/mL, as shown in Figure-2). The CT values were inversely proportional, using *B. melitensis* Ether strain CFU/mL values, as shown by the standard curve (Figure-2), where the correlation coefficient is equal to 0.998 and R^2^=0.0998 with high efficiency of 129.3% and y-intercept of 33.14 (Figure-2). The bcsp31-based RT-PCR used in this study was highly specific and sensitive compared with omp2 and 16S rRNA PCR, as reported by [34]. The authors chose the highly conservative bcsp31 gene that codes for a 31-kDa immunogenic protein for two reasons: (1) The presence of such a gene in all *Brucella* species [15] and (2) the small amplicon size (150 bp) produced by PCR when targeting such genes [35]. These two reasons may contribute in part to the improved sensitivity and specificity of the RT-PCR. The issues of lower sensitivity and specificity of the conventional PCR may be due to inhibitors in the clinical samples and limited analytical sensitivity of classic PCR that requires electrophoresis [36]. TaqMan RT-PCR targeting the highly conservative bcsp31 gene was used in this study to amplify *Brucella* genomic DNA in cattle serum samples (Figure-3). The IS711 conventional PCR targeting gene was used in this study to detect *Brucella* DNA in cattle serum samples at a size specific (839 bp) to the genus *Brucella*, as shown in Figure-4.

The PI, a single tool of accuracy, was estimated by adding the sensitivity and specificity of TaqMan RT-PCR and conventional PCR assays. PI summarizes the accuracy of both PCR methods in a single numeric value [18]. The facts support the superiority of the estimated PI (Table-3 and Figure-1) of TaqMan RT-PCR (182.3) compared to the conventional PCR (144.4), as evidenced by the superior DSe and DSp offered by RT-PCR and the lower false-negative (FN) and false-positive (FP) results.

All PCR techniques targeting the genomic DNA of *Brucella* in cattle sera agreed significantly with bacteriological isolation and typing at p<0.05. The estimated k agreement value for TaqMan RT-PCR was 0.825±0.042, while the corresponding agreement value for conventional PCR was 0.444±0.066 (Table-3). Kappa agreement values were classified by Landis and Koch [37] as follows: No agreement (<0), slight agreement (0-0.20), fair agreement (0.21-0.40), moderate agreement (0.41-0.60), substantial agreement (0.61-0.80), and almost perfect agreement (0.81-1). Based on this classification, TaqMan RT-PCR displayed a better agreement with bacteriological isolation and typing (almost perfect) over conventional PCR (moderate agreement). The possible reason for the aforementioned almost perfect agreement may be due to the bacteriology and PCR techniques’ capability to directly detect the *Brucella* organisms or their DNA in the target sample.

**Figure-5:**
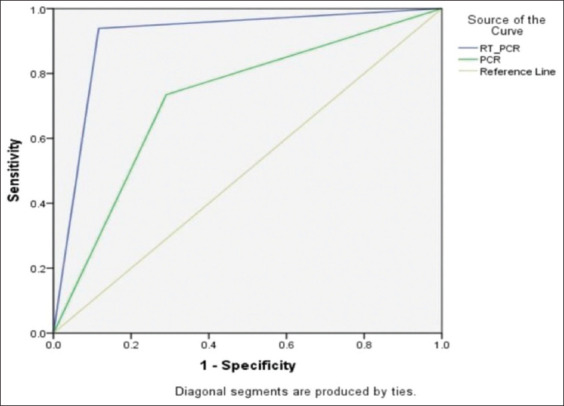
Receiver operating characteristic curves reflecting the diagnostic accuracy of TaqMan real-time polymerase chain reaction (PCR) versus the conventional PCR used to detect *Brucella* DNA in cattle sera.

The ROC curves show plots of sensitivity on the Y-axis against the FP rate on the X-axis and were created here (Figure-5) for evaluation of the results of RT-PCR and conventional PCR. The closer the curve toward the y-axis and the top boundary, the better the test performance. This improved performance based on the ROC curve is shown by the TaqMan based RT-PCR used in this study compared to the conventional PCR. The AUC can then be calculated as a single alternative accuracy indicator of both techniques [38]. The values of the AUC vary from 0.5 (no apparent accuracy) to 1 (perfect accuracy), with higher values over 0.5 indicating better test performance [38]. Under the field of this study, RT-PCR offers a higher accuracy (Table-3 and Figures-1 and 5) based on the ROC and AUC values (0.911) if compared with conventional PCR (AUC=0.722). RT-PCR revealed a better accuracy (91.3%) over conventional PCR (72.2%) as evidenced by the lower FP (10) and FN (6) results shown by the RT-PCT if compared with conventional PCR (FP=25 and FN=26), as shown in Table-3.

The PPV is the possibility that an animal that has tested positive is positive concerning the true diagnostic status. Predictive values are not inherent characteristics of a specific diagnostic test but are a function of DSe and DSp in a defined population at a given point in time. Predictive values are of great importance to field veterinarians for the interpretation of results. For example, a PPV of RT-PCR in this study, as shown in Table-3, is 0.9, meaning that an animal with a positive test result to RT-PCR has a 90% chance of indeed being infected and a 10% probability of testing falsely positive. In contrast, the PPV of conventional PCR is 0.74 (Table-3), meaning that an animal reacting positively to conventional PCR has a 74% chance of indeed being infected and a 26% probability of testing falsely positive. The predictive value of a positive result also has great importance for the veterinary services in charge of the management of control and eradication programs. The inverse of the PPV (i.e., 1/PPV) gives an idea about the amount of money spent in the culling of true- and FP animals detected by the surveillance activity. In other words, if the estimated PPV of conventional PCR, as in the case of this study, is 0.76, approximately two positive animals out of three are true positives, and the remaining is a FP.

The NPV is the possibility that an animal that has tested negative has a true negative diagnostic status. If the aim is to establish evidence for freedom from disease, the NPV is the most important measure. The highest NPV revealed by PCR techniques (Table-3) targeting *Brucella* DNA in cattle sera was achieved by TaqMan RT-PCR (0.92), followed by conventional PCR (0.7). The high NPV of RT-PCR indicates that, among those who had negative test results, the probability of being disease-free was 92% and 70% for TaqMan RT-PCR and conventional PCR, respectively. The reason for this finding may be attributed to the superiority of the TaqMan RT-PCR DSe over conventional PCR, as the NPV critically depends on DSe.

It is not practical to detect *Brucella* DNA in serum samples using conventional PCR, based on the determined PPV and NPV; serum samples are not the sample of choice for such a technique and vice versa is true for RT-PCR.

The above-mentioned diagnostic performance parameters issues noted for conventional PCR may indicate that a serum sample is not the material of choice for targeting *Brucella* genomic DNA for such a technique. The DSe, accuracy, and AUC issues of conventional PCR hinder the possibility of using it to diagnose brucellosis in serum samples, either as a screening or as a confirmatory test. The high FN results skew the measurements of any applied control policy, and high FP results lower its specificity and thus its ability to be used as a confirmatory assay.

Pearson correlation coefficient (r) was used in this study to assess the strength and direction of the relationship between RT-PCR, conventional PCR, and bacteriological results (Table-4). The Pearson correlation coefficient values ranged between +1 (positive correlation) and −1 (negative correlation), where the value of r=±0.9-1 is considered a very strong correlation, r=±0.8 or higher (strong correlation), r=±0.5-0.8 (medium correlation), r=±0.4 or lower (weak correlation), and r=0 for no correlation [39]. Subsequently, a strong positive correlation was noted (Table-4) between RT-PCR and bacteriological isolation and typing (r=0.826). Quite the opposite, a weak positive correlation was noted between conventional PCR and bacteriological results (r=0.444) from one side and between both PCR techniques from another side (r=0.443). The strong correlation which is estimated between RT-PCR and bacteriology may be attributed to the direct detection of *Brucella* organisms and their DNA in the serum samples by both methods and the better kappa agreement between them. These previous results concerning the superior performance of TaqMan PCR over the conventional PCR potentiate the capability of using such a technique as a rapid, accurate, and reliable tool to detect the genomic DNA of *Brucella* in serum samples (sample of choice) of cattle.

**Table-4:** Pearson’s r correlation coefficient of TaqMan RT-PCR, conventional PCR with the gold standard bacteriological isolation and typing.

PCRs correlation with bacteriological isolation and typing		PCR	Bacteriological examination	RT_PCR
PCR	Pearson correlation	----	0.444**	0.443**
	Sig. (two-tailed)	0.000	0.000
Bacteriological examination	Pearson correlation	0.444**	----	0.826**
	Sig. (two-tailed)	0.000	0.000
RT-PCR	Pearson correlation	0.443**	0.826**	----
	Sig. (two-tailed)	0.000	0.000	

** Correlation is significant at p=0.01 level (two-tailed). RT-PCR=Real-time polymerase chain reaction

As shown in Table-5, TaqMan RT-PCR showed superiority over conventional PCR as it was able to detect 95% (67/70) of *Brucella* DNA in the serum samples derived from serologically and bacteriologically positive animals of Group (1), while conventional PCR detected only 73% (51/70). These data are in agreement with those reported in other published papers where the same technique was used and the same gene was detected using the same TaqMan probe but serum samples of camels were used with Latent Class Analysis instead of bacteriological results as a gold standard [40]. These results of RT-PCR reflect the capability of this technique to detect the *Brucella* DNA in the serum sample perfectly and that may be attributed to the strong correlation and the almost perfect agreement of the TaqMan RT-PCR with the bacteriological results (gold standard) under the umbrella of this research.

**Table-5:** Molecular identification of *Brucella* DNA in serum samples of different groups of serologically and bacteriologically positive and negative animals.

Groups		RT-PCR		PCR	
	
		No. of positive	%	No. of positive	%
G1 (n=70)	(Positive bacteriology+positive serology)	67	95	51	73
G2 (n=28)	(Positive bacteriology+negative serology)	25	89	21	75
G3 (n=30)	(Negative bacteriology+positive serology)	7	23	17	56
G4 (n=56)	(Negative bacteriology+negative serology)	3	5	8	14

RT-PCR=Real-time polymerase chain reaction

TaqMan RT-PCR detected *Brucella* DNA in 89% (25/28) of the serum samples concerning Group 2 (serologically negative and bacteriologically positive animals), while conventional PCR detected 75% (21/28) in the same serum samples of Group (2). This finding may reflect the latent *Brucella* infection as a result of *in utero* or early postnatal infection among cattle of these groups. Animals can retain the infection for life and may remain serologically negative even after the first abortion or parturition [30]. Moreover, the long/variable incubation period of the disease results in FN reactions. Such cases can only be detected by PCR or classic bacteriological methods [12].

In Group 3 (bacteriologically negative but serologically positive animals), RT-PCR and conventional PCR detected 23% (7/30) and 56% (17/30) of *Brucella* DNA in the serum samples, respectively (Table-5). While in Group 4 (bacteriologically and serologically negative animals), RT-PCR and conventional PCR detected 5% (3/56) and 14% (8/56), respectively (Table-5), These results may be in part considered as FP results, but we cannot deny the issues regarding the sensitivity of bacteriological examination. As some animals may give negative cultural results, they are infected [18]. Reasons for this may be the condition of the submitted tissues, dead bacteria, samples with high contamination, or selection of an inappropriate or insufficient amount of tissue [18].

## Conclusion

Under the umbrella of the current research, the authors concluded that cattle serum samples are the samples of choice for detecting *Brucella* genomic DNA using the highly accurate, time-saving, and rapid TaqMan targeting the RT-PCR bcsp31 gene, based on the better diagnostic performance offered by such a technique over conventional PCR. It is also not technically or practically sound to use conventional PCR that requires optimization to detect the genomic *Brucella* DNA in serum samples of cattle.

The better diagnostic performance offered by the TaqMan RT-PCR in this study may be considered a step toward the possibility of using such technology in the direct differentiation between *Brucella*-infected and -vaccinated cattle immunized by smooth vaccines using cattle serum samples. This differentiation has been limited by most of the current serological tests.

## Authors’ Contributions

All the authors developed the concept and designed the study. NHA, EIMB, RII, MHMK, and NAS were in charge of performing the conventional PCR and serological diagnosis of the serum samples. NHA performed the statistical analyses. All the authors contributed to the drafting and revision of the manuscript and contributed to the manuscript writing. MAK and NHA were in charge of RT-PCR analysis. All the authors revised and approved the final manuscript.
